# Diagnostic Dilemma of a Neuroendocrine Tumour Complicated by Simultaneous Retroperitoneal Fibrosis and Carcinoid Heart Disease in a Perimenopausal Woman

**DOI:** 10.7759/cureus.86871

**Published:** 2025-06-27

**Authors:** Mohammad Adjmal Rummun, Nosamudiana Obazee, Mimi Leung, Vijeta Rummun, Anindya Shams, Zeeshan Tariq, Syed Nazarulla, Inamullah Khan

**Affiliations:** 1 Internal Medicine, Blackpool Teaching Hospitals NHS Foundation Trust, Blackpool, GBR; 2 Internal Medicine, East Lancashire Hospitals NHS Trust, Blackburn, GBR; 3 Diabetes and Endocrinology, Blackpool Teaching Hospitals NHS Foundation Trust, Blackpool, GBR; 4 Infectious Diseases, Blackpool Teaching Hospitals NHS Foundation Trust, Blackpool, GBR

**Keywords:** bilateral hydronephrosis, carcinoid heart disease, carcinoid syndrome, gastrointestinal neuroendocrine tumor, retroperitoneal fibrosis

## Abstract

Carcinoid tumours are rare, slow-growing neuroendocrine neoplasms that often remain asymptomatic until metastatic spread or the development of carcinoid syndrome. Carcinoid syndrome is characterised by flushing, diarrhoea, and bronchospasm due to the secretion of vasoactive hormones. These tumours commonly arise in the gastrointestinal tract but can also occur in other organs, namely, the lungs, genitourinary tract, and pancreas. Retroperitoneal fibrosis (RPF), a rare inflammatory disease, involves chronic inflammation leading to fibrous scarring and compression of surrounding structures like the ureters and blood vessels. Carcinoid heart disease secondary to fibrous valve thickening can also occur and causes high morbidity and mortality. This case report highlights a 52-year-old woman who developed a rare complication of RPF along with carcinoid heart disease secondary to a carcinoid tumour. Her symptoms, initially misdiagnosed as menopausal, included a four-year history of diarrhoea, uncontrolled hypertension, and flushing. She was admitted with abdominal pain, acute kidney injury, and bilateral hydronephrosis. Imaging and biochemical tests revealed a primary ileocecal carcinoid tumour with hepatic metastases, RPF causing ureteric obstruction, and elevated chromogranin A/B and urinary 5-hydroxyindoleacetic acid (5-HIAA) levels. Cardiac involvement included severe tricuspid regurgitation, pulmonary hypertension, and impaired left ventricular function, consistent with carcinoid heart disease. Management involved nephrostomy placement following failed bilateral ureteric stenting, octreotide infusions to prevent carcinoid crisis, and symptomatic control with lanreotide. The patient continues to receive multidisciplinary care from cardiology, urology, and oncology. This case underscores the complexity of diagnosing and managing carcinoid tumours with atypical presentations and rare complications like RPF.

## Introduction

Carcinoid tumours are uncommon, with an approximate annual incidence of 2.5 to five cases per 100,000 individuals and a prevalence of 35 per 100,000 [1]. They are slow-progressing neuroendocrine tumours that often stay asymptomatic until metastatic dissemination or manifestation of carcinoid syndrome [2]. Flushing, diarrhoea, and bronchospasm are hallmark features of carcinoid syndrome, driven by the production of vasoactive substances [3]. These neoplasms frequently emerge in the gastrointestinal tract but can also be found in the genitourinary tract, lungs, and pancreas [4]. Retroperitoneal fibrosis (RPF) is a chronic inflammatory condition characterised by persistent inflammation that results in fibrotic tissue formation and constriction of adjacent structures such as the ureters and blood vessels [5]. RPF occurring alongside a carcinoid tumour is a rare entity, with just a small number of cases described so far, the first appearing in the English literature in 1999 [5]. In a review of 824 cases of small bowel neuroendocrine tumours between 1985 and 2015, 16 patients were found to have developed RPF, suggesting it is an uncommon but noteworthy complication [6].

This case describes the rare association of RPF and carcinoid heart disease in a patient with carcinoid syndrome. The patient’s non-specific symptoms were initially misconstrued to perimenopausal changes, which led to a delayed diagnosis. Carcinoid syndrome is a rare and challenging condition to diagnose, and clinicians must maintain a high level of suspicion when presented with relevant symptoms.

This case report was previously presented as a poster presentation at the 2025 Society for Endocrinology (SfE) BES Conference on March 10-12, 2025.

## Case presentation

A 52-year-old Caucasian lady was referred by her general practitioner to the secondary care for deranged renal function. She presented with a three-week history of bilateral flank pain, nausea, and generalised weakness. Notably, she had chronic watery diarrhoea for more than three years, which had worsened recently. She also reported occasional flushing, which had been attributed to perimenopausal symptoms over the past few years.

**Figure 1:**
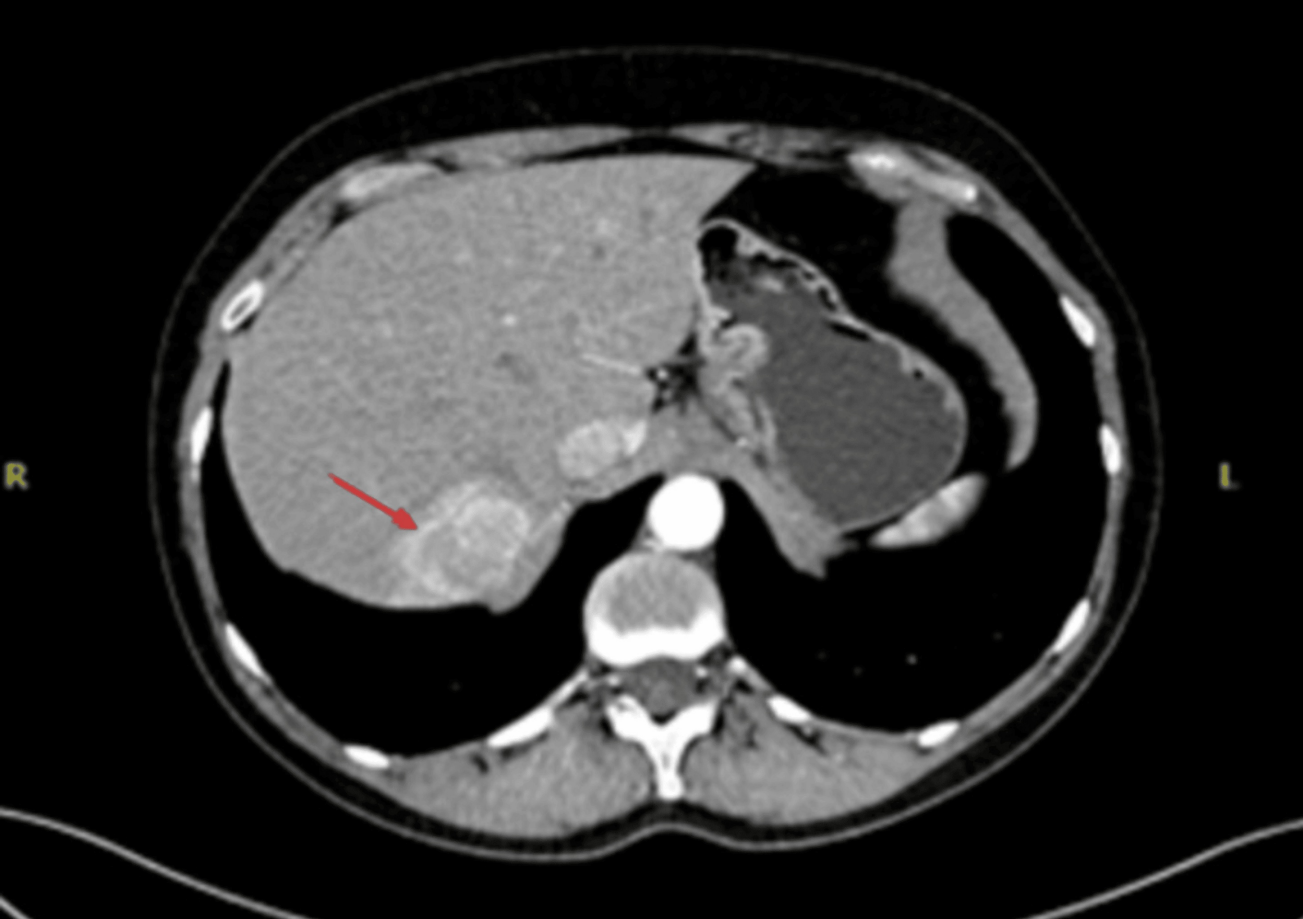
Initial CT of the abdomen reported multiple liver haemangiomas (red arrow).

Her past medical history included hypertension for which she was prescribed losartan and diltiazem. Previous investigations included a colonoscopy with biopsy a year ago, which showed ileocecal valve inflammation. Additionally, an ultrasound and CT scan of the abdomen revealed multiple liver haemangiomas (Figure 1).

Notably, she did not appear flushed on examination. The only positive findings were of a pansystolic murmur radiating throughout the precordium along with a right-sided flank tenderness.

Important negative findings were that auscultation of the chest did not reveal any wheeze, and no mass could be felt on palpation of the abdomen.

Investigations

Paraclinical investigations showed acute kidney injury (AKI) stage 2 (doubling of creatinine level) (Table 1), and the patient was admitted for evaluation of chronic diarrhoea and renal dysfunction.

**Table 1:** Renal profile showing stage 2 acute kidney injury. eGFR: estimated glomerular filtration rate.

Investigations (renal)	Baseline results (23/04/2024)	Admission results (15/08/2024)	Reference range
Urea (mmol/L)	6.2	7.8	2.5-7.8
Creatinine (μmol/L)	100	208	49-90
Sodium (mmol/L)	142	144	133-146
Potassium (mmol/L)	3.8	3.7	3.5-5.3
eGFR (ml/min/1.73 m^2^)	56	23	-

**Figure 2:**
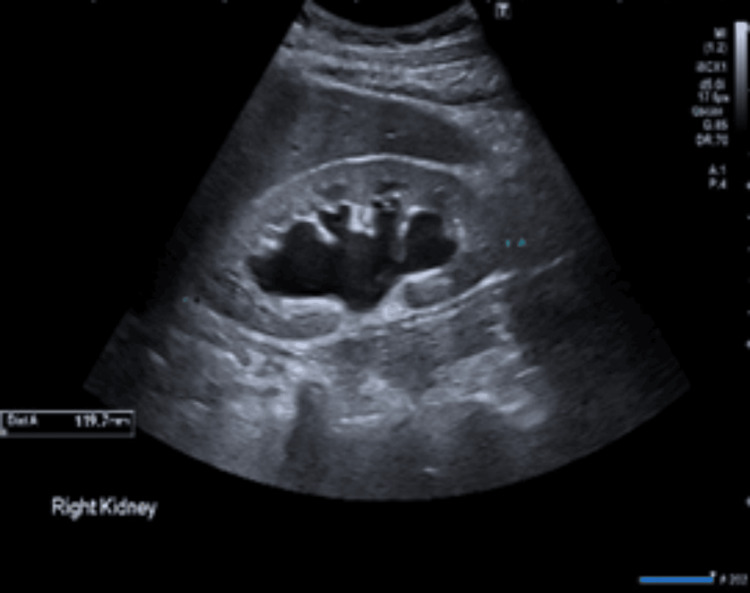
Ultrasound of the right kidney showing hydronephrosis.

**Figure 3:**
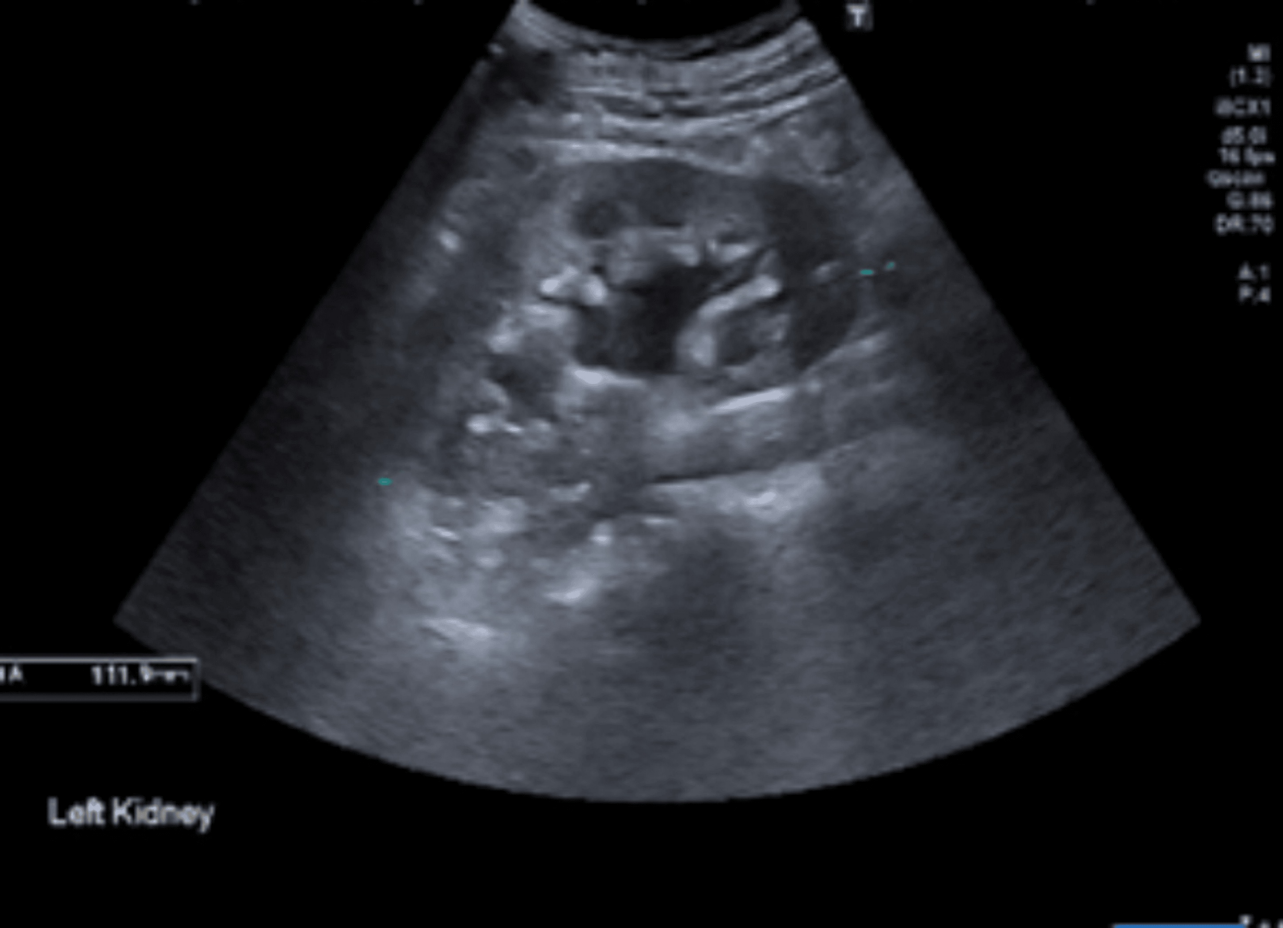
Ultrasound of the left kidney showing hydronephrosis.

**Figure 4:**
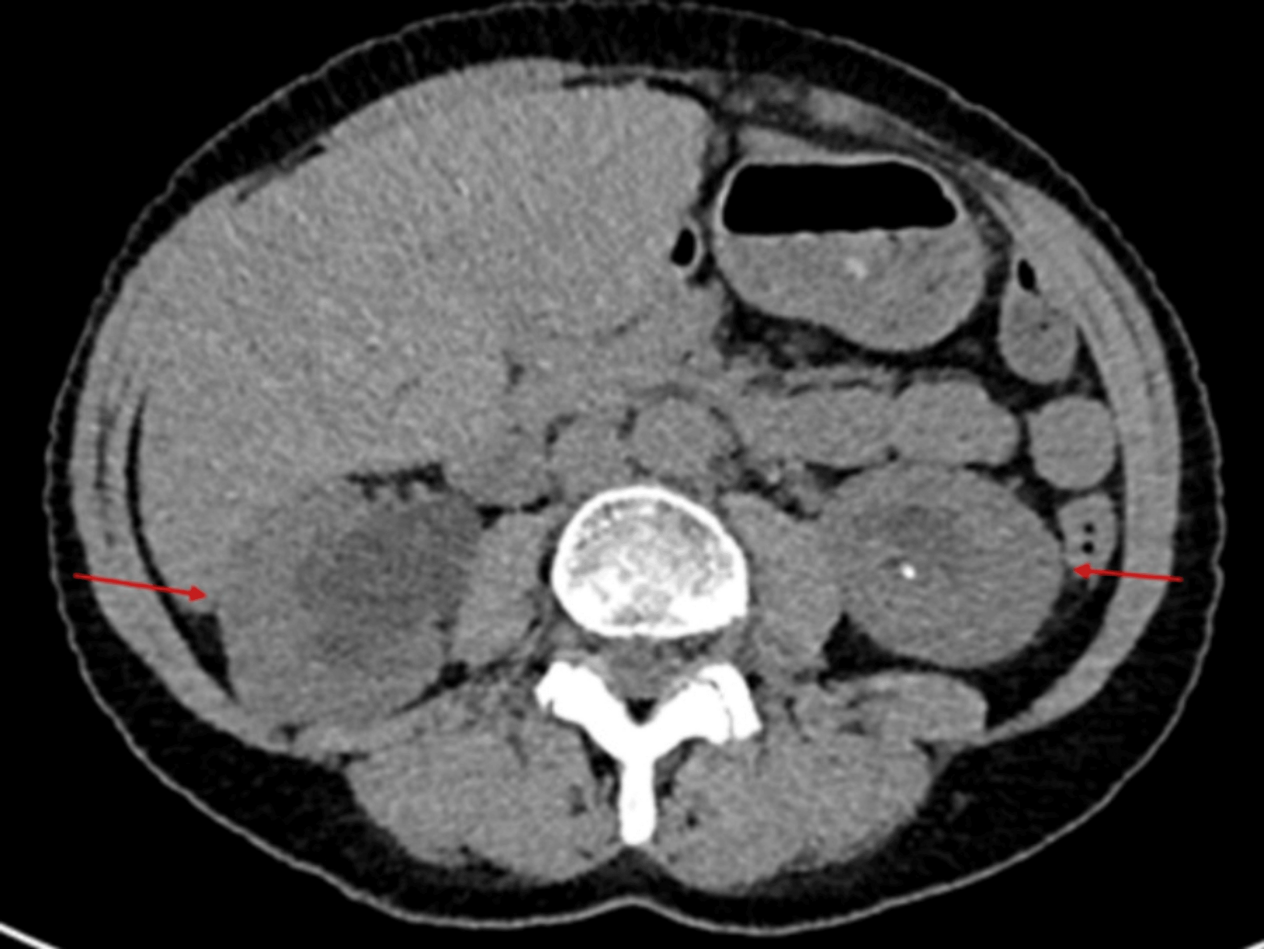
CT of the kidneys, ureters, and bladder showing bilateral moderate hydronephrosis (red arrows) secondary to retroperitoneal fibrosis.

Diarrhoea work-up meant investigations for thyroid profile, pancreatitis, coeliac disease testing, and faecal analysis, along with calprotectin and elastase were sent off, which all came back negative. Investigations for AKI through ultrasound (Figures 2, 3) and CT of the kidneys, ureters, and bladder (KUB) (Figure 4) revealed bilateral moderate hydronephrosis secondary to retroperitoneal fibrosis.

**Figure 5:**
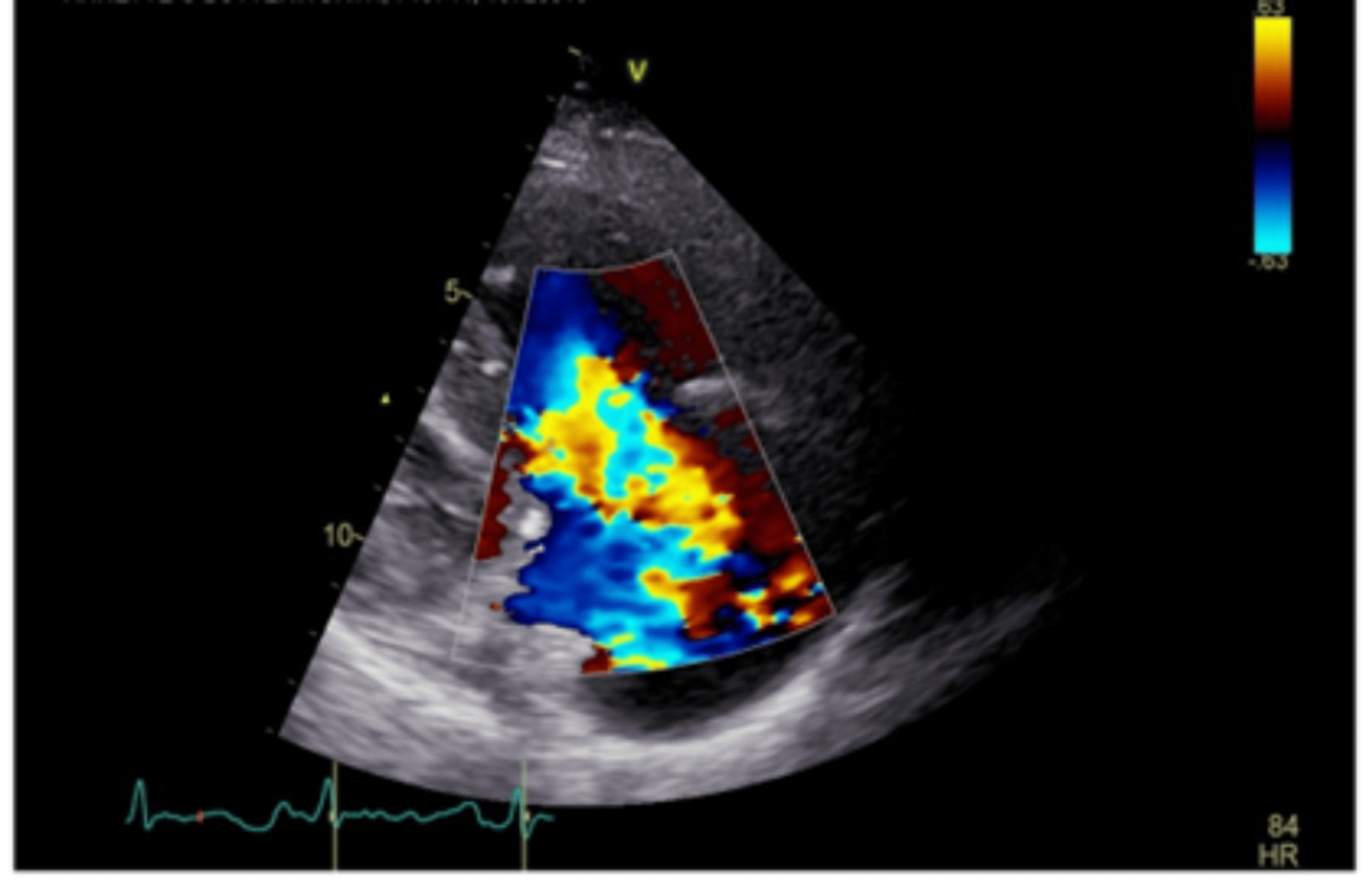
Severe tricuspid regurgitation on echocardiogram.

**Figure 6:**
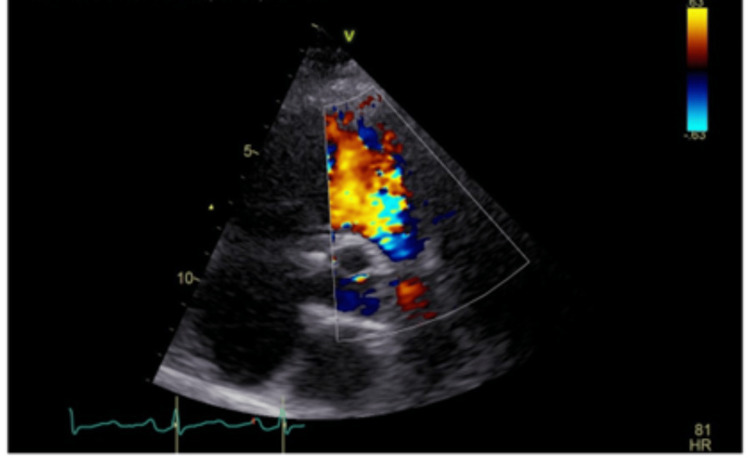
Severe pulmonary regurgitation on echocardiogram.

A cardiac echocardiogram done as part of initial investigations showed severe torrential tricuspid regurgitation (Figure 5), poor leaflet coaptation, moderate to severe pulmonary regurgitation (Figure 6), and a high likelihood of pulmonary hypertension. A subsequent ventilation-perfusion (VQ) scan ruled out pulmonary embolism.

Further investigations were conducted to assess for a neuroendocrine tumour, a possible cause of the combined right-sided valve disease, diarrhoea, and retroperitoneal fibrosis. A serum tryptase level was also ordered to exclude mast cell activation syndrome. Laboratory investigations are shown in Table 2.

**Table 2:** Laboratory investigations. VIP: vasoactive intestinal peptide; TSH: thyroid-stimulating hormone; 5-HIAA: 5-hydroxyindoleacetic acid.

Investigations	Result	Reference range
24-hour 5-HIAA (umol/day)	966	0-50
Chromogranin A (pmol/L)	3632	0-59
Chromogranin B (pmol/L)	1290	0-149
VIP (pmol/L)	16	0-29
Somatostatin (pmol/L)	33	0-149
Pancreatic polypeptide (pmol/L)	90	0-299
Gastrin (pmol/L)	14	0-40
Glucagon (pmol/L)	14	0-49
TSH (mIU/L)	2.18	0.3-5.0

**Figure 7:**
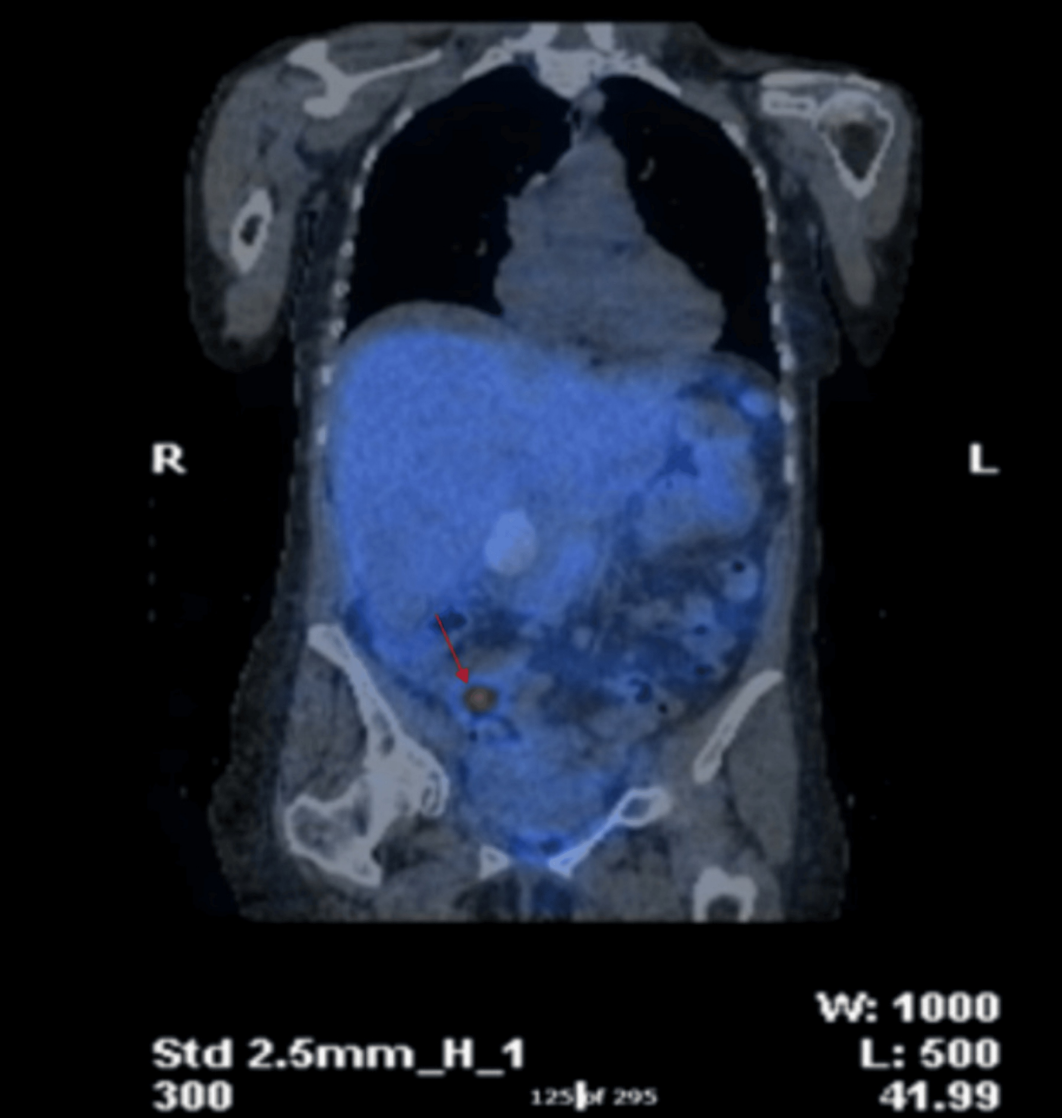
Octreotide scan with single photon emission computed tomography (SPECT) CT showing a 2.5 cm ileal mass (red arrow) with avid uptake of tracer.

**Figure 8:**
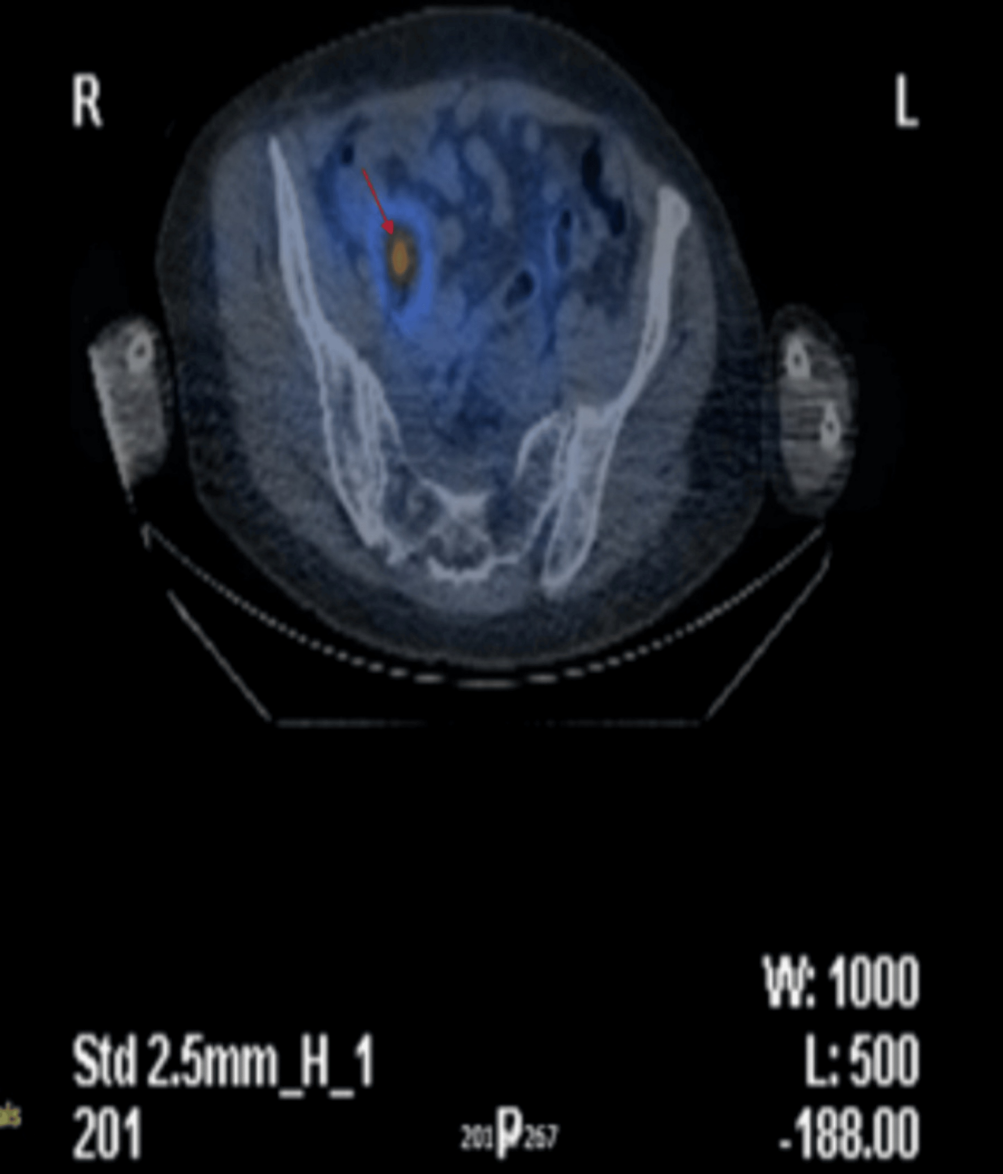
Octreotide scan with single photon emission computed tomography (SPECT) CT showing a 2.5 cm ileal mass (red arrow) with avid uptake of tracer.

**Figure 9:**
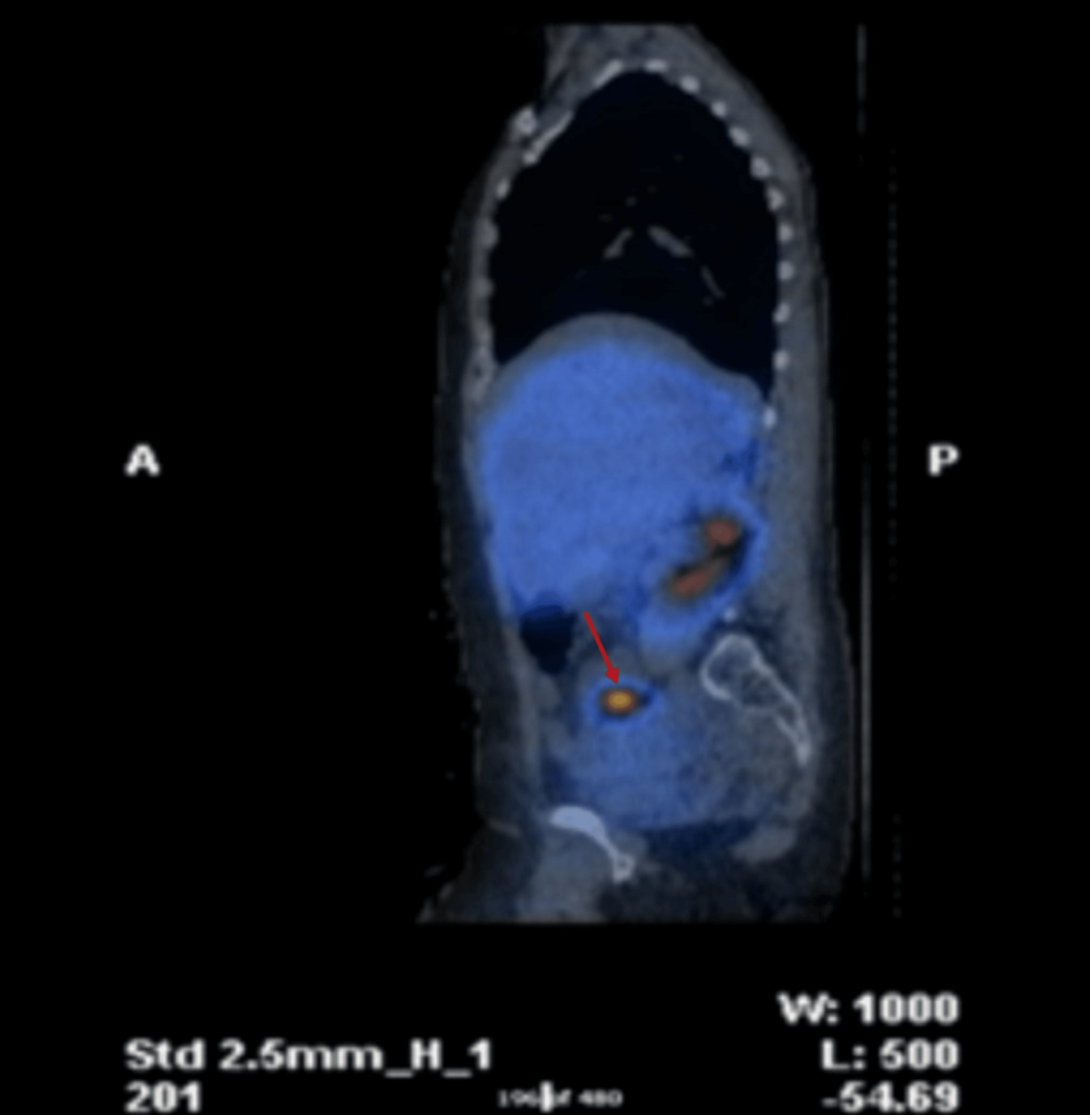
Octreotide scan with single photon emission computed tomography (SPECT) CT showing a 2.5 cm ileal mass (red arrow) with avid uptake of tracer.

An octreotide scan with single photon emission computed tomography (SPECT) CT revealed a 2.5 cm ileal mass showing avid uptake of tracer and exhibiting somatostatin II, III and V receptor activity (Figures 7-9), confirming the diagnosis of a small bowel neuroendocrine tumour. The combination of increased biomarkers, valvular abnormalities, and imaging results supported the diagnosis of carcinoid syndrome.

Treatment

Management was conducted through a multidisciplinary team (MDT) approach involving endocrinology (for titration of somatostatin analogues), urology (to monitor and manage hydronephrosis), cardiology (for valvular and heart failure surveillance), and oncology (for potential radio/chemotherapy).

Symptomatic relief was initiated with octreotide (50 mcg three times a day for 10 days) and lanreotide (120 mg subcutaneous (SC) every 28 days).

Bilateral hydronephrosis was initially managed with ureteric stents, but as renal function did not improve, bilateral nephrostomies were performed to prevent further kidney damage. A preoperative octreotide infusion (12.5 mcg/hr for 12 hours) and continued infusion for 24 hours postoperatively were administered to prevent carcinoid crisis, as per the UK & Ireland Neuroendocrine Tumour Society (UKINETS) guidelines [7].

Outcome and follow-up

It is too early to assess the treatment's full effect. The patient is currently being followed by the neuroendocrine tumour (NET) oncology department and will be considered for a liver biopsy to confirm the nature of the hepatic lesions.

**Figure 10:**
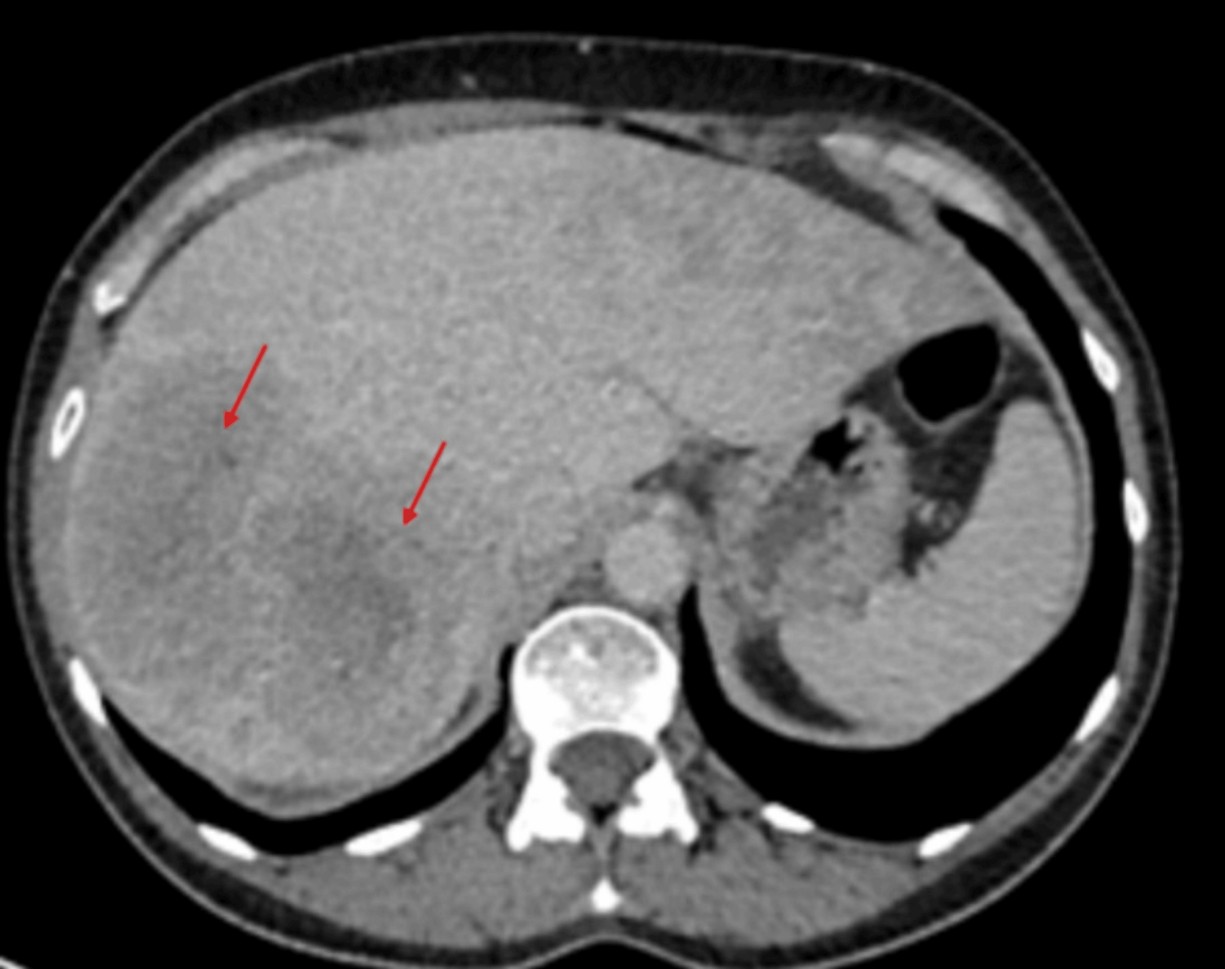
Initial CT of the thorax/abdomen/pelvis on 28 August 2024 showing multiple liver lesions (red arrows).

**Figure 11:**
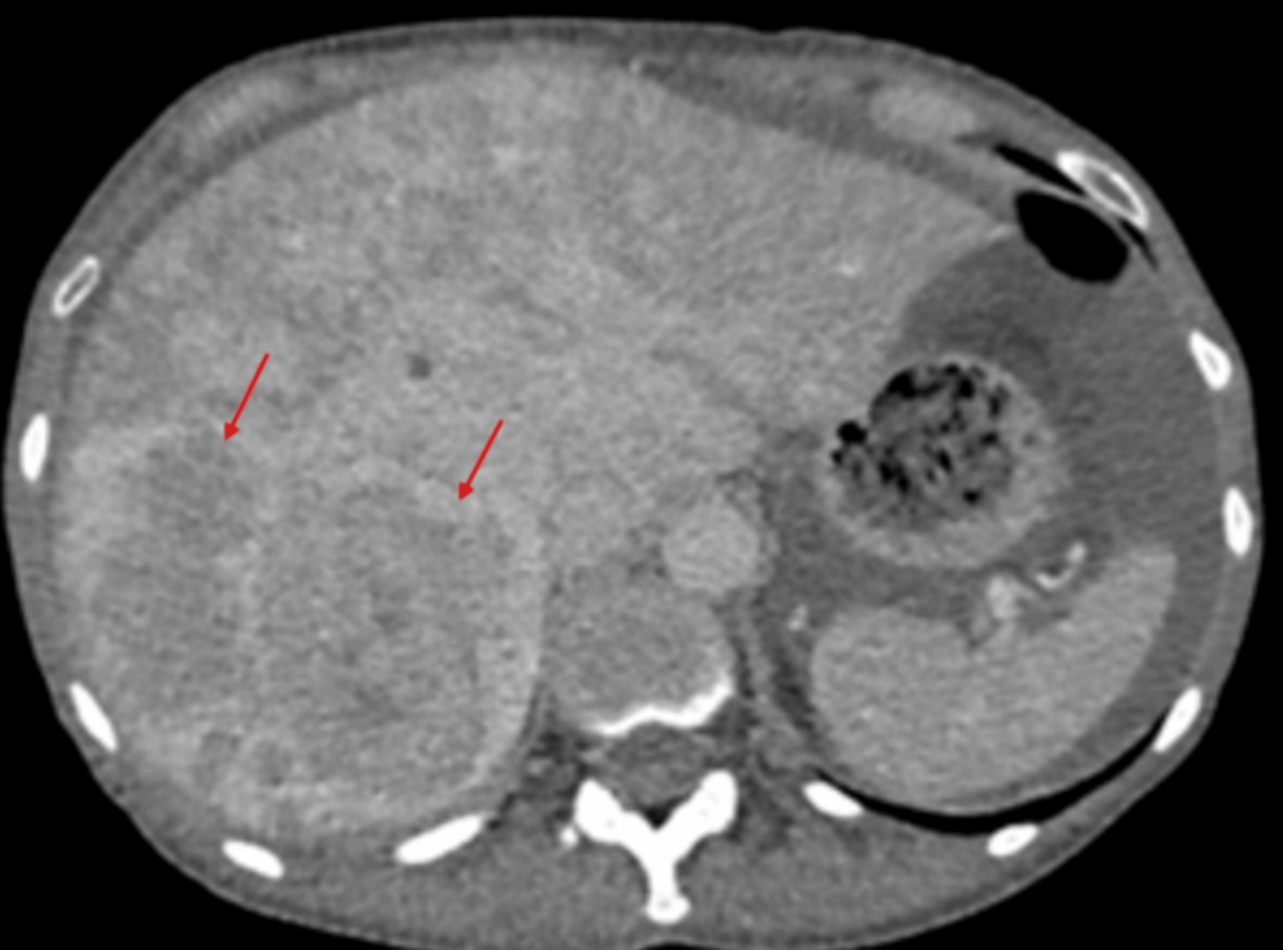
Repeat CT of the thorax/abdomen/pelvis on 7 February 2025 showing liver lesions (red arrows) with no evidence of gross interval changes.

**Figure 12:**
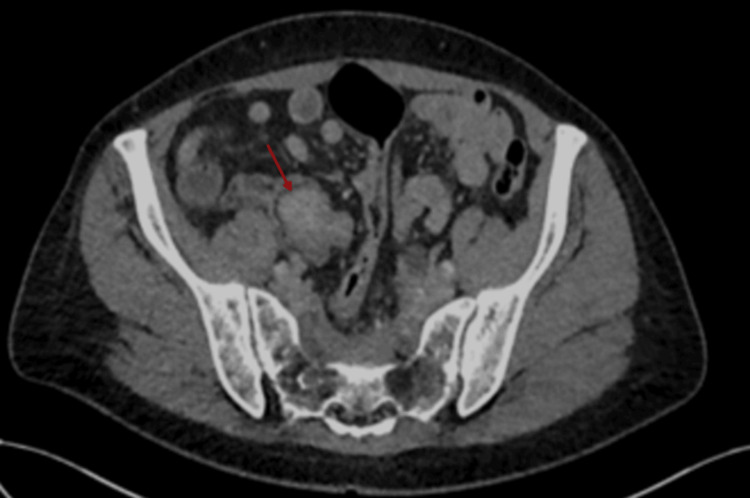
Initial CT of the thorax/abdomen/pelvis on 28 August 2024 showing retroperitoneal fibrosis and an enhancing lesion at the ileocecal junction (red arrow).

**Figure 13:**
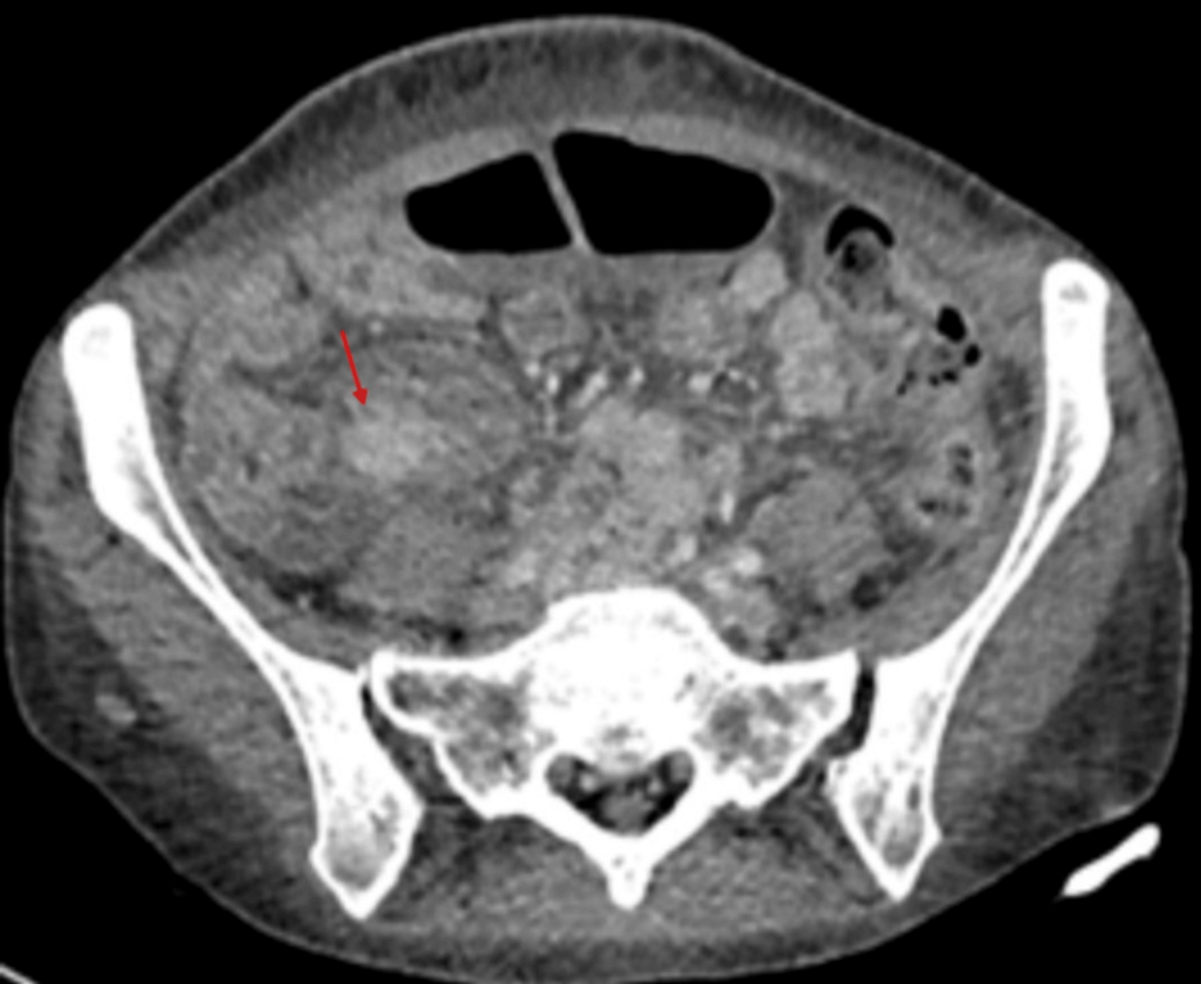
Repeat CT of the thorax/abdomen/pelvis on 7 February 2025 showing stable retroperitoneal fibrosis and a mild increase in size of the enhancing lesion at the ileocecal junction (red arrow).

After being on lanreotide for six months, an echocardiography was repeated, which showed features similar to the previous one, with impaired left ventricular systolic function of 45-50%. Severe tricuspid regurgitation, along with moderate to severe pulmonary regurgitation, was again demonstrated on the repeat echocardiography. She is being regularly reviewed and assessed in the heart failure clinic to monitor progression and has been started on furosemide for symptomatic relief. A 24-hour urine collection for 5-hydroxyindoleacetic acid (5-HIAA) was also requested and came back at 647 umol/day (0-50), which showed a reduction from the initial figure. Further treatment has been offered to reduce the disease burden. Otherwise, from a more clinical perspective, the patient now complains of significantly less diarrhoea, less flushing, but has lost 16 kilograms in the last six months despite being on supplemental nutrition. A repeat CT of the thorax, abdomen, and pelvis showed stable retroperitoneal fibrosis, no evidence of gross interval changes of the multiple liver lesions and a mild increase in size of the enhancing lesion at the ileocecal junction (Figures 10-13). To conclude, it is worth noting that despite the fact that the main symptoms have subsided, long-term complications, namely, carcinoid heart disease and retroperitoneal fibrosis, have had limited reversibility from the treatment as demonstrated by the subsequent echocardiogram and CT scan findings.

## Discussion

The non-specific symptoms in this case, including those associated with perimenopause, made the diagnosis of a neuroendocrine tumour challenging.

The development of retroperitoneal fibrosis in association with a carcinoid tumour is an uncommon event, documented thus far in only a limited number of case reports [5]. The association between these two pathologies shows that any post renal obstructive uropathy, which could potentially be caused by retroperitoneal fibrosis, should raise the suspicion of a carcinoid tumour due to its ability to secrete neuroamines and neuropeptides, which lead to inflammation and hence fibrosis in the retroperitoneal space.

A review of small intestine neuroendocrine tumour cases carried out between 1985 and 2015 highlights that retroperitoneal fibrosis is a rare but notable complication that cannot be overlooked in the context of carcinoid syndrome [6].

Mesenteric fibrosis, another potential complication, may lead to intestinal obstruction, oedema, and ischaemia. With the increasing knowledge of biopathological processes, new targeted therapies to treat or prevent fibrosis are being investigated, namely, somatostatin analogues [8], tamoxifen [9], and erlotinib [10], amongst others.

However, most of these therapies are still under clinical trials or early stages of study, and as such might be useful in future. In this case, where the diagnosis of retroperitoneal fibrosis was made on hydronephrosis secondary to extrinsic ureteral compression (considered pathognomonic), a more urgent approach in the form of surgical intervention (double J stent then requiring nephrostomy) was indicated to prevent further renal damage. Otherwise, surgery remains the mainstay treatment for retroperitoneal fibrosis (lysis/bypass).

The occurrence of carcinoid heart disease together with retroperitoneal fibrosis in this case of carcinoid tumour is what makes this case a very rare and distinct one.

Considering the above, we think it is imperative to include carcinoid tumours among the causes leading to retroperitoneal fibrosis and to possibly evaluate the presence of these tumours in labelled idiopathic cases of retroperitoneal fibrosis.

## Conclusions

Carcinoid tumours should be considered in perimenopausal women presenting with additional symptoms such as lethargy, flushing, diarrhoea, and abdominal pain. Retroperitoneal fibrosis and carcinoid heart disease are rare complications of carcinoid tumour and carcinoid syndrome. Carcinoid tumours should be considered in the differential diagnosis of idiopathic retroperitoneal fibrosis. It is important to emphasise that early recognition of these symptom clusters can aid diagnosis and avert irreversible complications such as cardiac and retroperitoneal fibrosis.

Carcinoid heart disease requires a high index of suspicion, particularly in cases of right-sided valvular involvement and heart failure symptoms without evidence of pulmonary disease or pathology. This case highlights the critical role of a multidisciplinary team approach in managing carcinoid syndrome to facilitate early diagnosis and improve patient outcomes.
